# How Sleep Activates Epileptic Networks?

**DOI:** 10.1155/2013/425697

**Published:** 2013-09-12

**Authors:** Peter Halász

**Affiliations:** National Institute of Clinical Neuroscience, Lotz K. Straße 18, Budapest 1026, Hungary

## Abstract

*Background*. The relationship between sleep and epilepsy has been long ago studied, and several excellent reviews are available. However, recent development in sleep research, the network concept in epilepsy, and the recognition of high frequency oscillations in epilepsy and more new results may put this matter in a new light. *Aim*. The review address the multifold interrelationships between sleep and epilepsy networks and with networks of cognitive functions. *Material and Methods*. The work is a conceptual update of the available clinical data and relevant studies. *Results and Conclusions*. Studies exploring dynamic microstructure of sleep have found important gating mechanisms for epileptic activation. As a general rule interictal epileptic manifestations seem to be linked to the slow oscillations of sleep and especially to the reactive delta bouts characterized by A1 subtype in the CAP system. Important link between epilepsy and sleep is the interference of epileptiform discharges with the plastic functions in NREM sleep. This is the main reason of cognitive impairment in different forms of early epileptic encephalopathies affecting the brain in a special developmental window. The impairment of cognitive functions via sleep is present especially in epileptic networks involving the thalamocortical system and the hippocampocortical memory encoding system.

## 1. Introduction

The robust activation of epileptic interictal and ictal activity in NREM sleep is well known and valid for almost all types of epilepsies. Different aspects of relationship between sleep and epilepsy were addressed in several excellent reviews [1–4]. During development of the last years, several new aspects have been elaborated contributing to understand better the activation of interictal and ictal epileptic phenomena by sleep.

The most important issues among them are as follows. (1) Sleep physiology has revealed neuronal networks governing wake-sleep alternations and cyclic changes during night sleep. Nowadays we see better the interrelationship between the sleep-wake circuitry and its multifold relationship with the different epileptic networks. (2) In the microstructure of sleep certain dynamic key points have shown to be associated with epileptic activation identified within the system of cyclic alternating pattern (CAP) correlating with reactive slow wave events. (3) One of the most important among recent discoveries is the exploration of the high frequency range of EEG and the recognition the relationship of this phenomenon with epilepsy and slow wave sleep. (4) Recognition of epileptic encephalopathies have shown that within a certain developmental window epileptic activity-usually during sleep-takes over the conduction in important physiological systems, and by the principle of “firing together-wiring together” epilepsy is hijacking physiological functions. (5) Underlying point 4 the key mechanisms by which interictal epileptic activity during slow wave sleep is interfering with cognitive functions have been increasingly studied.

*Research Background*

*Sleep Research*

*Anatomy, Neurochemistry, and Dynamic Interaction of Wake and Sleep Promoting Circuits.* The existence of two antagonistic systems promoting sleep and wake state was assumed already in 1930 by von Economo [5] based on autopsy findings of victims of European encephalitis letargica pandemia. He proposed that region of the hypothalamus near to the optic chiasm should contain sleep promoting, and the posterior hypothalamus wake promoting neurons. 

From the forties of the last century the concept of an ascending “arousal system,” maintaining wakefulness, in the brainstem of animal and human brain, became more and more clear [7]. The several transmitters (acetylcholine, noradrenalin, serotonin, catecholamine, histamine, and orexin) serving detailed functional properties of this system were also step by step revealed [8–10]. 

**Figure 1:**
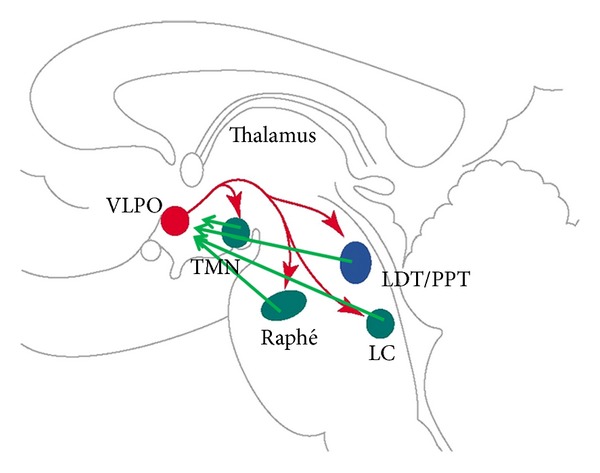
Brainstem-hypothalamic sleep-wake system. VPLO represents sleep promoting neuronal assemblies exerting inhibitory influence (red) on arousal promoting ascending pathways. Arousal system originating from different nuclei working with different transmitters (LC = locus coeruleus, adrenergic, raphe nuclei, serotoninergic, TMN = tuber mamillare, histaminergic, LDT/PPT: = lateral dorsal tegmental/pedunculopontine, cholinergic) exerts reciprocal inhibitory influence on VLPO sleep promoting neurons. (modified after Saper et al., 2001 [6]).

At the turn of the 20/21 century, Saper and coworkers [6] and others [12] showed that the ventrolateral preoptic area (VLPO and extended VPLO) send GABA-ergic and galanin-ergic inhibitory impulses to all the brainstem nuclei harbouring the ascending pathways of the arousal systems and keep firing throughout the whole NREM sleep, providing the substrate of the “sleep system” with opposite function to the “wake system.” Later it turned out that nuclei of the arousal system also exert inhibitory effect on the “sleep promoting” preoptic neurons. “When VLPO neurons fire during sleep, they would inhibit the arousal system cell groups thus disinhibiting and reinforcing their own firing. Similarly when arousal neurons fire at high rate during wakefulness, they would inhibit the VLPO, thereby disinhibiting their own firing” [6]. This reciprocal relationship is nowadays more or less accepted as the elementary hypothalamic “sleep switch” module underlying alternations of sleep and wake state (Figure 1).

*NREM Physiology Underlain by the Special Burst-Firing Working Mode of the Thalamocortical System. *The inhibition of the arousal systems by the extended VLPO system has a further consequence, namely, the liberation of the thalamocortical system, because during wake state the arousal systems exert tonic cholinergic inhibition on the thalamocortical system. Liberation of the thalamocortical system is reflected by widespread development of spindling, delta activity, and slow (below 1 Hz) oscillation as characteristic by-products of the complex interrelationship of cortical (pyramidal cells), thalamic (relay neurons), and reticular (nucleus reticularis thalami (NRT) constituents of the system).

During wakefulness, the system works as a relay centre which faithfully conveys input from the outer world towards the cortex. This is executed by the so-called “tonic activity” of the network reflected by desynchronized EEG.

 When we go to sleep, a cascade of events starts, and the working mode of the thalamocortical system is going to change toward an excitatory-inhibitory cycle, namely, burst-firing mode in which the nucleus reticularis thalami periodically inhibits firing of the thalamic relay nuclei, and this sequence is reflected on the cortex either as spindles or as deltas depending on the level of membrane polarisation of the relay cells. Thalamic structures isolated from NRT do not show oscillatory behaviour, while the NRT produce spindling even after isolation from the rest of the thalamus [13].

A further player of slow wave sleep: the slow oscillation below 1 Hz had been described in the nineties (in cats by Steriade et al. 1993 [14] and in humans by Achermann and Borbély [15]). The cortical nature and widespread presence throughout the cortical mantle were proven by several studies [14, 16, 17]. The slow oscillation consists of a depolarizing and hyperpolarizing phases, namely “up” and “down states.” While the up state is characterized by rich neuronal and synaptic activity, and contains high frequencies, during the down state the cortical network is globally disfacilitated. 

Studies of NREM sleep rhythms clearly showed that they are closely interconnected appearing in coalescence. The depolarized part (up-state) of the slow oscillation below 1 Hz envelops delta rhythm and spindling together with gamma and ripple degree fast activity [20, 21]. Combinations of spindles, K-complexes, and delta activity with fast rhythms are the product of interplay between cortical and thalamic structures within the thalamocortical system.

*Dynamic Structure of NREM Sleep Fuelled by Reactive Phasic Changes Related to Arousal Influences. *Exploration of the so-called “microstructure” of NREM sleep [22] revealed that stages of sleep (standardized by Rechtschaffen and Kales) consist of continuous fluctuations which are kept in motion by phasic changes called “microarousals” (reflected by EEG changes, autonomic signs, and muscle activity without awakening). These reactive phasic events (elicitable by sensory stimuli and assumable appearing as a reaction to some external (or internal) stimuli) create abundant fluctuations, lend flexibility to the sleep structure by which microfluctuations led to the development of macrofluctuations (stage shifts), and ensure a flexible connection between the sleeper and the surrounding world.

In the mid-eighties, Terzano and coworkers discovered a hitherto not recognized long-term cyclicity during NREM sleep related to sleep perturbations, namely cyclic alternating pattern (CAP) [23].

**Figure 2:**
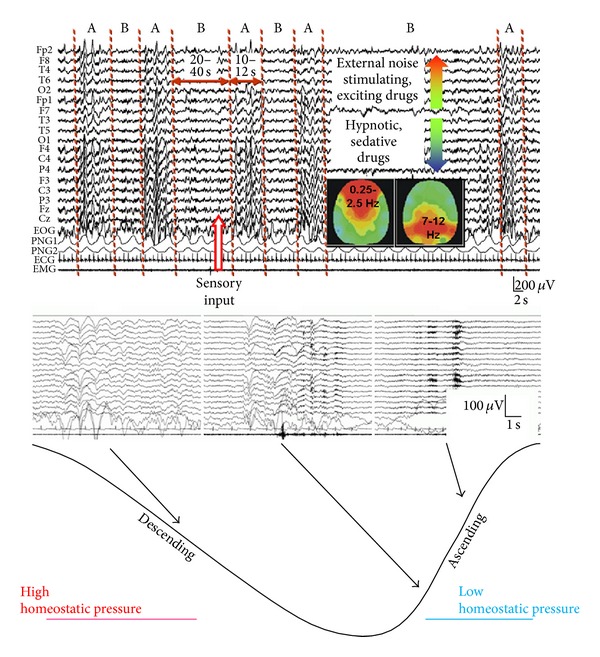
Schematic features representing the Cyclic Alternating Pattern (CAP) phenomenon. Above: alternation of activated (a) and background (b) episodes with characteristic parameters. In the middle: electrographic features of three kinds of A phases (A1-A2-A3). Below: schematic representation of the sleep cycle with descending and ascending slopes. A1 type phasic activation is associated with the high homeostatic pressure periods of sleep cycles, while A2 and mainly A3 type phasic activities are associated with low homeostatic pressure periods of sleep cycles.

The CAP cycle consists of two phases: a phase A and a phase B. Phase A is mostly identical with the phasic activation events (see below for more details), while phase B is characterized by the background level of the sleep stage (Figure 2). Sensory stimuli in phase B are able to elicit the phase A pattern. In CAP, the arousal-dependent phasic events are arranged in complex pseudoperiodic assemblies. The mean period time between two A phases is about one minute. The average phase A duration is 10–12 sec, while the average length of phase B is 20–30 sec. According to the type of activation reached by phase A, three categories can be differentiated. 

Phase A1 type comprises exclusively synchronization patterns (alpha in stage 1, sequential K-complexes in stage 2 and superficial stage 3 and reactive slow wave sequences in stages 3 and 4). It is identical with the synchronization-type microarousals [24].

On a slightly higher level of arousal, phase A2 type is composed of microarousals preceded by synchronization composed by K-complexes or slow waves, followed by either sigma or alpha and delta stretches. 

On the highest level of arousal, the phase A3 type will be a microarousal without slow waves. This is identical with the traditional desynchronisational microarousal [25] (Figure 2).

The percentage of CAP time in NREM sleep (CAP rate) is age related. The CAP rate is high in very early infancy (up to 100% of NREM sleep in the newborn in the form of “trace alternant”). It declines to 44% among teenagers and diminishes to 25–30% in young adults. It then increases to an average of 54% in older age groups. The CAP rate correlates negatively with the subjective evaluation of the quality of sleep (the higher the CAP rate, the poorer the quality of sleep). The CAP rate is also increased by external noise and lowered by prolonged sleep deprivation. Stimulating and arousing drugs increase and hypnotic/sedative drugs decrease the CAP rate. The power spectral analysis of CAP phenomena revealed [26] that CAP corresponds to periods in which frontal dominant very slow delta activity groups together a range of different EEG activities. Distribution of the different phase A subtypes proved to be different across the sleep cycles. On the descending (D) slope and especially in the first cycles phasic activity is less frequent and characterised by synchronisation type and sleep-like slow wave answers (A1 type), associated with mild autonomic perturbations. On the ascending (A) slope phasic activity is more frequent, and both the EEG morphology and the concomitant autonomic changes correspond more to the conventional A2 and A3 type phasic activation [27].

**Figure 3:**
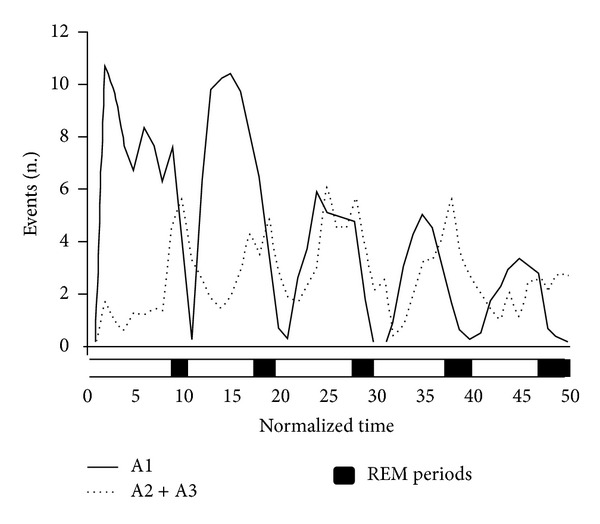
Different distribution of CAP A1 and A2-3 phases across night sleep. The distribution of A1 phases follows the classical pattern of slow wave decline from evening to morning predicted by the behaviour of the S-process of Borbély. The distribution of A2-3 phases has a different course; their amount do not decrease from evening to morning and show recurrent peaks before and during REM sleep (modified after Terzano et al., 2005 [11]).

CAP is an integrated part of the NREM sleep slow wave activity. Since 30–40% of sleep time is spent in CAP A phase (CAP rate) and A1 phase is more than 60% of all the CAP sequences, reactive slow activity in the form of A1 phase is a considerable amount of sleep slow waves. CAP A1 rate in NREM sleep undergoes a characteristic significant exponential shape reduction from the beginning to the end of sleep [11]. It is parallel with the behaviour of the slow wave activity course across the night and with the homeostatic S process of Borbély [28] furthermore congruent with the dampening course of K-complex rate during sleep [29]. At the same time, rate of phase A2-3 showed quite different distribution with cyclic peaks before the REM periods on the ascending slopes of cycles, without any dampening during the course of sleep (Figure 3).

The tuning of sleep-wake balance probability lies in the relationship between the reciprocal antagonistic wake promoting ascending arousal systems and sleep promoting VLPO system, and CAP sequences reflect the balance between sleep and wake promoting systems. When VLPO system exerts GABA-and galanin-ergic inhibitory influence and keeps firing in a certain rate during sleep, the inhibitory influence of the arousal system dissipates. When a phasic arousal influence is arriving, the VLPO system became transitorily weakly inhibited, but if the arousal influence does not continue, the VLPO system became again liberated fuelling the backinhibition of the arousal system producing (quasi rebound) sleep phenomena (CAP A1 phase). This situation is valid especially when on the descending slopes of first cycles; the VLPO system is prevailing (during high homeostatic pressure), and the arousal system is weak. During the third part of sleep on the ascending slopes of the cycles, the situation is different: the VLPO system is less active in inhibiting the arousal system; therefore, the arousal impulses activate easily the cortex, and these phasic arousals achieve more prominent arousal in the form of CAP A2 and A3 responses driving the sleeper toward more superficial vigilance states. 

*NREM Sleep Homeostasis Serves Plastic Changes and Recuperation of Cognitive Functions. *In the last 10–15 years, local aspects of homeostatic regulation receive more and more attention. Beyond the classical knowledge of previous wake state proportional delta power increases during the next night, and the frontal preponderance of sleep slow activity and further the frontal dominance of the recovery increase after sleep deprivation, and dominant hemisphere preponderance was emphasized in several studies [30, 31]. Increasing lines of evidence support the role of slow wave sleep in human frontal cognitive functions [32]. Beside the frontal localization in homeostatic regulation associated with certain cognitive functions, different kinds of “use-dependent” increase of regional slow wave activity have been registered after particular functional usage. Extensive sensory stimulation of one arm before sleep led to an increase of delta power in the opposite hemisphere over the somatosensory arm area [33]. An opposite intervention: immobilization of the arm caused a local reduction of delta power in the same localization [34]. In the light of these experiments delta power of sleep seems to depend on the amount of afferent activity or in other experiments on the degree of learning-related change in synaptic strength before sleep [32]. Stickgold and coworkers [35] have confirmed that some learning (texture discrimination task) occurs only after a night of sleep. Sleep-deprived subjects fail to improve on texture discrimination even after two recovery nights. The phylogenetic aspect of sleep-dependent learning came also in the focus of research. Early life sensory deprivation in animals reduces sleep slow wave activity [36].

So, recent studies have shown that procedures presumably leading to local plastic changes in the cerebral cortex can result in local changes in slow wave activity during subsequent sleep, or in other words, the homeostatic process is use dependent and related essentially to cognitive activity.

The association of slow waves with cognitive processes is supported by the sleep findings in clinical disorders with mental decline. In normal senescence, parallel with the decay of mental activity, the sleep delta activity and the amount of delta rebound after sleep deprivation decreases [37]. Disorders damaging frontal lobes with consequent mental decline show in the same time sleep delta reduction. Alzheimer's disease and chronic alcoholism are good examples for this [38]. The same situation is detectable in sleep apnoea where apnoeic periods do not allow the development of delta sleep; consequently, cognitive functionsmay show important impair reflected by hypoperfusion of frontal lobes [39]. Chronic insomnia with deficient sleep delta activity also may impair cognitive functions [40, 41].

After summing up the most relevant new aspects of sleep research in the next chapters we will apply this knowledge to obtain new view points to understand more precisely the activation effect of NREM sleep in epilepsies. 

*Epileptic Networks.* During the last 5–10 years due to several new data, a slow but decisive change seems to be developing in thinking about “generalized” and “focal” epilepsies. These research data clearly show that “generalized” epilepsies are not really generalized and “focal” epilepsies are not focal. Therefore the classical dichotomy of partial and generalized epilepsy became more and more meaningless, giving place to a unifying network concept. The so-called “generalized” epilepsies involve a bilaterally represented large cerebral system related to wide cortical association areas, namely, the thalamocortical system. “Focal” epilepsies are also not strictly localized to a geometrical “focus” but involve more or less wide sometimes bilateral (e.g. temporal and occipital epilepsies) regional circuitries. Concerning this kind of “network epilepsies”—although the seizure onset might origin consequently from one or more relatively restricted areas—the whole mechanism is more complex and determined by several influences and interrelationships, involving and transforming (in early onset) the functioning of physiological systems. Idiopathic epilepsies are embedded in functional systems of the brain and our knowledge about epilepsy is always dependent on the actual level of understanding in neuroscience, especially in neurophysiology.

The development of cognitive neuroscience led us to understand better the working modes in the higher order cortical areas [42–46].

Research of the last decade showed that all the mental processing serving cognitive functions, from simple to sophisticated ones, and the elaboration is executed instead of serial (sequential) processing by the strategy of “parallel distributed processing” [47]. 

Each module taking part of processing may serve in a flexible way different functional assemblies switching their composition according to the computational demand. The modules have no singular importance; only the pattern of cooperation among them is decisive. 

Hierarchical features between brain areas are not able to explain the complexity and speed of binding the cerebral players to each other in cognitive functioning. Beyond hierarchy and connectivity (spatial dimension) another mechanism (in the time dimension, by temporal synchrony) recognized only at the middle of the nineties became the main candidate to explain binding. This temporal synchrony seems to work through synchronization of cerebral rhythms, especially by gamma oscillations. 

Several lines of evidence demonstrated the appearance of gamma oscillation when higher elaboration was evoked by a sensory stimulus and when a cognitive task was processed. These findings provided lines of evidence for local oscillatory ensembles related to gnostic and cognitive elaboration in the cortex. A second step was to reveal that neuron ensembles apart from each other and even across to cerebral hemispheres come together in time transiently by gamma-frequency synchronization. This was evidenced firstly in the visual system [48] but later turned to be general principle working in several systems [49].

These above summarized features (parallel processing and binding by synchrony in gamma frequencies) delineate the working mode of physiological networks determining higher order processing in the brain. 

Due to the multifold aspects revealed by the contemporary neuroimaging, neuropsychological, quantitative EEG (Q-EEG), and other sophisticated approaches of epilepsies, even when we can point out a certain area the resection of which renders the patient seizure free, nowadays it became clear that epileptic disorders reside not only in certain cortical spots or within geometrical mensuration but also extend in a more wider network. 

The most important argument in favour of the network oriented view comes from the nature of epilepsy itself. It resides in preformed physiologically meaningful structures, with complex special situation, and possesses remote influences. Furthermore just because the characteristic increased level of excitability, it is very sensitive to triggering inputs. So epilepsy should be conceptualized in a more extended network than the seizure onset zone to which the surgery oriented approach mainly attached.

Nowadays we got more and more lines of evidence that epilepsy along with the physiological interconnections oversteps the artificial procrustean bed of anatomical lobes [50–52]. This is also an argument for conceptualizing epilepsies related to physiological networks instead of anatomical localizations.

The strict localization-related epilepsy concept has also a major drawback. The so-called generalized epilepsies are not fitting into it. Since more and more lines of evidence support the view that idiopathic generalized epilepsies are not really generalized and their characteristics are explained by the epileptic disorder of the thalamocortical system, using the working mode of the system in NREM sleep, this kind of epilepsy has its explanation also in a physiological network turned to become epileptic (system epilepsies). 

Neuronal networks in the cortex generate several distinct oscillatory bands, covering frequencies from <0.05 Hz to >500 Hz. Oscillators of different bands couple with shifting phases and give rise to a state of perpetual fluctuation between unstable and transient stable phase synchrony. The resulting interference dynamics are a fundamental feature of the global temporal organization of the cerebral cortex. 

Collective behaviour of neurons is established through synchrony. Events that can be integrated over time by the target neurons are synchronous. Oscillatory coalition of neurons to form population synchrony may have a time window from hundreds of milliseconds to many seconds. Neuron assemblies are formed as transient coalitions with mutual interaction of discharging neurons [53]. 

Extension of EEG records to the high frequency oscillation (HFO) range revealed new vistas in epilepsy-related electrical activity and also shed more light to the properties of epileptic networks [54].

Two types of HFO have been distinguished: (a) ripples (R) with slower frequency (80–160 Hz) as physiological activity and (b) fast ripples (FR) with higher frequency (200–500 Hz) as a pathological activity, associated with epilepsy, described in experimental epilepsy models and in epileptic patients both with mesiotemporal and cortical epilepsies [55–57]. Concerning interictal HFO, the majority of them were associated with epileptiform sharp waves in time with the spike and not with the after-coming slow wave [58–60]. Interestingly HFO activity is maximal during slow wave sleep (up states) [61–64].

 A manipulation that decreases HFO reduces the likelihood of seizures [65]. Increased FR ratio correlates with hippocampal cell loss and synaptic reorganization in temporal lobe epilepsy [63].

It seems to participate in epileptogenesis, being present already during the “silent period.” A manipulation that decreases HFO reduces the likelihood of seizures. Therefore HFO and especially FR seem to be an essential player in epileptogenesis and in the electrographic phenomenology of epilepsies; furthermore it opens new diagnostic possibilities concerning the epileptic networks. 

New spike and seizure functional MRI (fMRI) data mapping the irritative and seizure network in epilepsy provides a new functional anatomy of epileptic networks.

The systematic studies of Gotman and coworkers in MNI from 2003 and others [66–73] detecting epileptiform discharges and seizures by fMRI have led to several very important conclusions. They showed that spike discharges are related to either local blood oxygenisation level dependent (BOLD) positive or remote BOLD-positive/BOLD-negative signals. Even one spike may induce, beside local activation, remote consequences either in terms of excitation or depression of functions. 

Generalized spike and wave type discharges show thalamic BOLD positive and diffuse cortical mixed signal with predominance of BOLD negativity. “Similarly” secondary bilateral “synchrony” type discharges show important diffuse cortical BOLD-negative components. Thus BOLD activation might make the network structure of irritative activity in the epileptic brain measurable. 

Ictal studies were possible only limitedly due to movement artifacts when movements are prominent. However, when possible, BOLD activity may show in a noninvasive way the seizure pacemaker zone and the propagation network as well. These studies clearly proved that interictal and ictal discharges have much more widespread influence on brain functions than we conceptualized before. What is more, the exerted remote effect of spike and seizure activity is organized into complex activation/deactivation patterns that should be still more understood and probably will contribute to new functional anatomy epileptic networks.

Recently with group analyses of the individual interictal BOLD changes in different types (temporal and frontal lobe and posterior quadrant epilepsies) of epilepsy, Fahoum et al. [74] from the Gotman group were able to demonstrate common metabolic changes characteristic for the group that may not be apparent in images of the individual patients. For example, in temporal lobe epileptic (TLE) patients the largest activation cluster was in the cingulate gyri (ipsi- and contralateral), and the second cluster by volume included the insula, amygdala, and anterior hippocampus ipsilateral to the focus. 

The positive BOLD activation was joined by common negative BOLD activation in the contralateral inferior parietal lobule and bilaterally in the posterior cingulum and precuneus (mostly in default mode network (DMN) structures). In patients with frontal lobe epilepsy (FLE) group analysis resulted in BOLD positive activation in the bilateral cingulum, contralateral cerebellum, ipsilateral frontal operculum, medial thalamus, genu, and posterior limb of the internal capsule.

Thus they confirmed that interictal discharges recorded from the scalp EEG may represent only a fraction of broader events that involve widespread brain areas despite their focal appearance. The group analyses proved that unique interictal networks can be related to different types of partial epilepsies.

Based on these studies we can assume that certain networks characteristic to the epilepsy type are under special “discharge load” with probable altering of the function of the involved structures, even during interictal periods without seizures.

*Sleep Activation in Different Epileptic Networks*

*Thalamocortical Epileptic Network Underlying the Electroclinical Phenomenology of Epilepsies Hitherto Classified as Idiopathic Generalized Epilepsy (IGE).* Under this heading, different groups of epilepsies are classified. The common clinical features are absences, multilocular myoclonic jerks, or generalized tonic-clonic seizures appearing with characteristic age dependency and the lack of initial focal symptoms (however thre are different lines of evidence for the frontal seizure origin both in experimental works [75] in clinical case histories).

From a syndromatological point of view, the following types were delineated: (1) childhood absence epilepsy (CAE), (2) juvenile absence epilepsy (JAE), (3) myoclonic absence epilepsy (MAE), (4) eyelid myoclonia with absences (EMA), (5) perioral myoclonies with absences (PMAE), (6) juvenile myoclonic epilepsy (JME) with jerks without loss of consciousness and generalized tonic-clonic seizures (GTC) seizures, and (7) IGE with awakening GTC seizures (EGMA).

The EEG shows in interictal state certain variations of bilateral frontal dominant spike and wave discharges. Ictal EEG pattern is bilateral extended spike-wave discharges (absence), multiple spikes and waves (myoclonic seizures of JME), and repetitive spiking in the tonic, then slowing down to packages of spike-waves and alternating with suppressed periods (generalized tonic-clonic seizures). 

We have evidence, provided by several studies, where during interictal spike-wave discharges a transient cognitive impairment (TCI) is detectable [76].

Neuroimaging did not show structural cerebral alterations studied by routine protocols, however in JME patients frontomedial structural changes were demonstrated by histological [77] and by MRI morphometric methods [78]. The disorder has an inheritance probably caused by a variation of multigenetic constellations not yet thoroughly revealed with separate endophenotypic characteristics of the seizure, EEG, and other features (like photosensitivity). 

The working mode of the system by which the spike pattern develops is proved to be the same that works during NREM sleep opposed to waking and REM state (see in the previous chapter in more details). This working mode is characterized by “burst firing” when both cortical and thalamic neurons produce excitation alternating with inhibition or disfacilitation in high synchrony. The pacemaker of inhibition executed on the thalamic relay cells is the thalamic reticular neurons. Electrophysiological studies revealed a very complex thalamocortical circuitry with multiple transmitters and special membrane properties involving cortical pyramidal, thalamic relay, and thalamic reticular neurons producing intermittent recurrent excitation and inhibition behind the characteristic bilateral spike-wave pattern. 

*Relationship between NREM Sleep and IGE Symptoms. *There is a close relationship between vigilance level and expression of spike-wave paroxysms. Spontaneous paroxysms are promoted by transitory decreases of vigilance level during awake state[79, 80], after awakening, after lunch, in evening sleepiness, during boring tasks or situations, experimental depression of reticular arousal functions [81], and after sleep deprivation. Spontaneous paroxysms are inhibited by a sudden increase in vigilance [82, 83], arousals (calling by name), and experimental stimulation of the reticular arousal system. This relationship stems from the common “burst firing” working mode of the thalamocortical system sharing by a mechanism that sets into motion both in shifts toward slow wave sleep and in spike-wave pattern.

**Figure 4:**
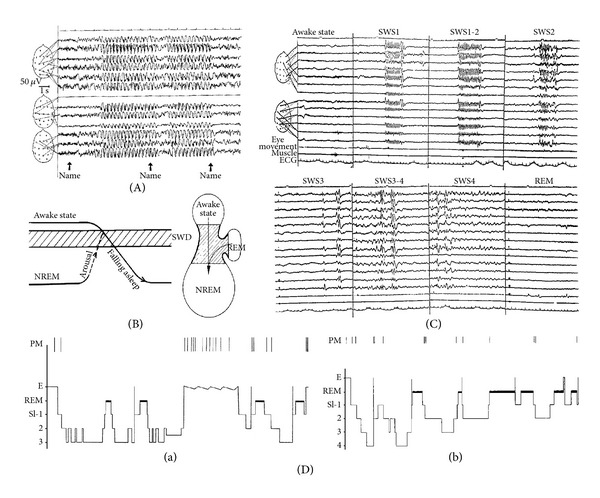
Composite figure demonstrating the association of ictal type of spike-wave paroxysms with transitory periods between awake and NREM sleep. (A) shows that absences with generalized spike-wave pattern can be elicited by arousing stimulus (calling by name), while the same arousal blocks the pattern by increasing level of vigilance. (B) Schema of the optimal level for absences reachable either by arousal from light sleep or falling asleep. (C) and (D) The distribution of spike-wave activity across the sleep stages. Absence type ictal activation appears exclusively in the transitional periods between awake/NREM and REM/NREM sleep. Attentional awake state usually and REM sleep always block absences. (D) Distribution of absences (indicated by perpendicular lines) across sleep cycles in night sleep in absence epileptic patients.

However, the fact that spike-wave activation in the form of absence-like 3 Hz paroxysms occurs selectively in transitional periods (between slow waves sleep and wakefulness and between slow wave and REM sleep) and that spike-wave pattern is absent in REM sleep both in humans [78, 79, 84–86] (Figure 4) and animals [87, 88] and is present only in distorted groups during deep slow wave sleep needs explanation. Studies analyzing this relationship have shown that not only the level of vigilance differs but activation in these transitional periods is closely connected with sudden oscillations of vigilance attached to the so-called phasic events of sleep. Spontaneous paroxysms (with or without clinical manifestations) have been associated with arousal-dependent phasic events preceded by K-complexes and/or slow waves [89, 90]. With sensory stimulation these dynamic changes could have been experimentally elicited and studied [90]. Association of generalized spike-wave pattern in IGE with sleep instability in NREM sleep can be measured by the CAP phenomenon, the frequency of which is proportional with sleep instability. Sleep EEG analysis of primary generalized patients [91] showed significant prevalence of spike-wave paroxysms during CAP as compared to NCAP periods (68% versus 32%), 93% of all the spike-wave patterns occurred in CAP were found in the reactive phase A. In sleep EEG analysis of JME patients [92] spiking rate was significantly higher in CAP A phase compared to NCAP and showed strong inhibition in CAP B phase. The link between EEG microarousal phenomena and spike-wave paroxysms is in apparent contradiction to the association of spike-wave pattern and sleep-like bursting mode of the thalamocortical system. To solve this contradiction we should take into consideration that most of the evoked phasic events during the dominance of the bursting mode show the features of sleep response. They contain clear-cut synchronisational slow wave sleep elements (single or serial K-complexes, slow wave groups) occurring in the same form as in the spontaneously appearing counterparts. Each phasic activation during slow wave sleep seems to evoke a regulatory rebound shift toward sleep, which seems to be the best activator of the oscillatory mode of thalamocortical network and the spike-wave mechanisms as well [84, 90].

There is another aspect in which NREM sleep and absence seizures share important features, namely, the global decrease of cortical activity during both absences and NREM sleep. Absences with 3 Hz synchronized spike-wave pattern are composed by two distinct components. The underlying events during the “spike” component proved to be unequivocally a pronounced glutamatergic burst discharge both in cortical and thalamic relay cells. The “wave” component was previously viewed as summated inhibitory postsynaptic potentials attributed to GABA-ergic inhibitory process in pyramidal cortical neurons. Later Steriade [20] showed that instead of inhibition a “disfacilitation” (decreased neural activity) is present during the “wave” component.

Transcranial doppler (TCD) and single photon emission computed tomography (SPECT) studies in experimental animals [93, 94] and in humans [95] showed cortical decrease and thalamic increase of blood flow during absences. On functional magnetic resonance imaging (fMRI) studies thalamic structures showed positive BOLD activation, while over wide cortical fields patchy negative BOLD activation has been observed [66].

Similar features proved to be true for NREM slow oscillation (as it was described in the first part). Steriade et al. [14] discovered that beside delta activity a slower oscillation (<1 Hz, generally 0.5–1.0 Hz) exists, and this slow oscillation plays role in grouping of delta waves and spindles during deep NREM sleep, with mechanisms essentially cortical in origin. The slow oscillation consists of a prolonged depolarizing phase (up-state), followed by long-lasting hyperpolarization (down-state). The up-state shows dense excitatory and inhibitory synaptic activity, while the down-state is characterized by cessation of synaptic barrages (disfacilitation). The Steriade group provided ample evidence that slow oscillation involves the cortex widely, opposed to faster oscillations originating in more restricted circuits.

*NREM and IGE Absences Share Common Physiological Circuits. *Both in deep NREM sleep and in absences the cortical activity is reduced in certain (mainly frontal) areas. In absence the loss of contact with the outer world may have a twofold reason, because (1) the bursting mode interrupts the continuous flow of information from our surrounding to the cortex, (2) the disfacilitation during the “wave” component involving the cortical association areas decreases the possibilities of cortical elaboration. The sensory information flow is impaired in both conditions. Sensory stimuli have an awakening effect in sleep and a disruptive effect in absence seizures too. Sleep induction promotes absences and awakening inhibits both sleep and absences.

So the functional neuroimaging, neurophysiologic, and clinical data became recently highly congruent, pointing to the thalamocortical network as a common substrate of NREM sleep and IGE. Therefore the slogan of Steriade: “sleep and epilepsy are bedfellows” is really very witty here. Spike-wave discharges of IGE represent the epileptic exaggeration of the bursting mode of the thalamocortical system. Therefore the inducement or shift toward NREM sleep promotes the manifestations of IGE. The well-known activating effect of sleep deprivation and sleep per se is probably related to the same mechanism [96].

Since the thalamocortical system can be influenced by other cortical and brain stem systems the epileptic disorder may originate from several routes (e.g., as gen-related channelopathies). As yet we do not know which one is realized in the human phenotypes. Several experimental and genetic models exist demonstrating this multiplicity by which the same system can be activated. 

*Idiopathic Focal Childhood Epilepsies (IFCE), Landau-Kleffner Syndrome (LKS), and Electrical Status Epilepticus in Sleep (ESES) Continuum.* Within this spectrum of epileptic disorders the first group, namely, the IFCE itself, consists of a spectrum to which all the idiopathic focal childhood epilepsies belong. Nowadays we see Idiopathic Focal Childhood Epilepsies as a spectrum of epileptic disorders being the most frequent, genetically based nonlesional epilepsy in childhood [97]. However, the clinical boundaries of the syndromes, which belong to this spectrum, are still in evolvement, the following diagnostic criteria seem to be already solid: (a) normal neurological examinations, (b) normal intelligence, (c) normal neuroimaging, (d) family history of seizures, especially benign types, (e) monotypic seizures, stereotyped in clinical manifestation, (f) frequent occurrence in sleep, (g) seizures are easily controlled with antiepileptic drugs, and (h) beginning age determined, with onset rarely before 3 years or after the age of 16-17 years and with spontaneous, age-dependent resolution, principal EEG features include normal background activity, spikes with characteristic morphology and localization, resembling to focal spike-wave discharge, activation during sleep, and in a part of patients generalized SWD. 

There is a large diversity in the topography of spikes. Patients with benign focal epileptiform discharges (BFED) frequently have bilateral-independent or bilateral synchronous discharges. The spikes in idiopathic centrotemporal epilepsy (ICTE) may be ipsi- or contralateral to the symptomatogenic side. Furthermore, BFED are frequently multifocal. The most frequent combinations are bilateral-independent or synchronous centrotemporal or occipital discharges. The centro-temporal and occipital spikes are also frequently seen in combination, and some patients have more than two spike foci in a recording. 

All these observations tend to change the concept of focal epilepsy towards a more widespread genetically based condition of increased cortical excitation with shifting predominance. The epileptic dysfunction is not localized to a circumscribed small area, but it is imbedded in a broader network of the associative cortex. Probably the most relevant approach is the concept of Doose et al. [98] stressed also by Panayiotopoulos et al. [99], who assumed behind these floating features a “seizure susceptibility disorder” related to certain “hereditary maturational impairment.”

**Figure 5:**
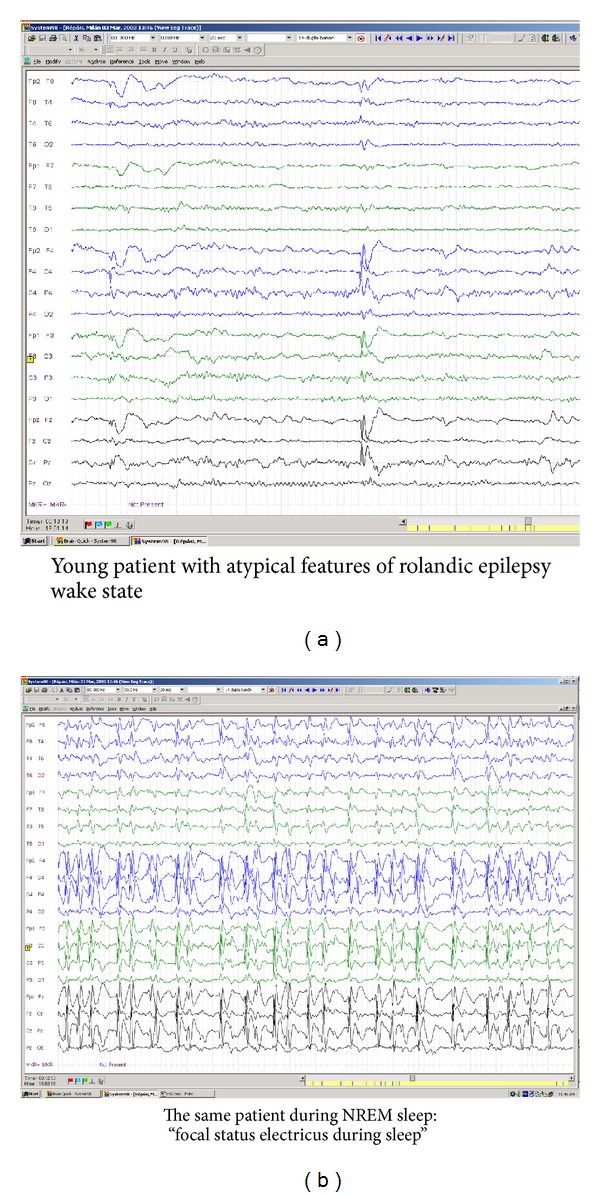
Rolandic epilepsy with atypical features: progressive cognitive impairment and focal status epilepticus like activation of interictal discharges during NREM sleep.

In the last years the most frequent form of IFCE, Rolandic epilepsy with centro-temporal spikes turned to be less “benign” as it was assumed before [100, 101], and the described so-called “atypical” variants paved the way to find the continuum between them and LKS and ESES. Nowadays we seeseveral variations of patients seemingly belonging to the focal benign childhood epilepsies. These patients show in awake state focal interictal discharges, but they may have abundant serial focal (pseudogeneralized) discharges, not rarely focal status electricus in NREM sleep and have more cognitive impairment during their long-term course (Figure 5).

LKS is a childhood disorder occurring in previously normal children, characterized by the loss of language skills, acquired verbal auditory agnosia, multifocal spikes, and spike-wave discharges mainly localized over the centrotemporal regions, continuously or subcontinuously during sleep. Epileptic seizures (usually rare), behavioural disorders, and hyperkinesias are observed in about two-thirds of patients. “The prognosis is favourable, with remission of seizures and EEG abnormalities before the age of 15 years” [102]. No evidence of associated focal brain lesions has been documented. Deonna and Roulet-Perez [103] in their recent review emphasized that “Landau-Kleffner is now seen as the rare and severe end of a spectrum of cognitive-behavioural symptoms that can be seen in idiopathic (genetic) focal epilepsies of childhood, the benign end being the more frequent typical rolandic epilepsy.”

At the early stage of the disorder unilateral IEDs are more common, resembling discharges seen in IFCEs, localised to a wide area around the Sylvian fissure. Bilateral “generalized” SW is also common. Later bilateral focal discharges are prevalent in roughly homologous areas. 

ESES is a rare condition characterised by the coexistence of the following aspects: (1) ESES pattern in NREM sleep occurring in at least 85% of NREM sleep and persisting on three or more records over a period of at least one month; (2) cognitive impairment, in the form of global or selective regression of cognitive functions; (3) motor impairment (ataxia, dyspraxia dystonia, or unilateral pyramidal deficit); (4) rare epileptic seizures (particularly in the period of ESES) with focal and/or generalised seizures (uni- or bilateral clonic, GTC, and complex partial seizures or epileptic falls tonic seizures never occur). The EEG abnormalities disappear around puberty, and the epilepsy shows a benign outcome too, but the cognitive impairment is not completely reversible in all cases, and some residual impairment remains. LKS and ESES are separate entities, but the two conditions show a wide overlap.

These epilepsies are characterized by the abundance of regional epileptiform discharges in sharp contrast with the rare and in several cases lacking seizures. The nature and severity of interictal cognitive symptoms are closely related to localization within the network and amount of epileptic interictal discharges. 

In this spectrum disorder activation in sleep has a special significance.

In IFCE NREM sleep characteristically increases the occurrence rate, amplitude, and distribution field of interictal epileptiform discharges(IEDs) [104]. Some studies found that “maximum spike/min ratios were related to slow sleep stages, especially delta sleep, and in general to the first cycle” [105]. A relationship between spikes and spindle sleep had been shown long ago [97, 106, 107].

In LKS NREM sleep has a strong activating effect. The transition from LKS to ESES during long-term clinical and EEG followup has been described [108–110]. Usually REM shows no discharges, and in all cases described no generalized GSW were present during REM sleep. Methohexital studies performed by Ford et al. [111] showed that one hemisphere is “driving” the other in bilateral discharges.

In ESES the NREM sleep activation is the most characteristic feature, in the form of GSW 1.5–2 Hz discharges presenting continuously across all sleep stages, beginning immediately with the start of NREM sleep [112]. The pattern of cognitive impairment may differ from patient to patient; the proportion of speech dysfunction is also variable and the type of impairment seems to depend on the localisation of the field exhibiting generalized spike-wave (GSW) discharges [113–116]. There is a close temporal relationship between ESES and the neuropsychological deterioration and parallels between the duration of ESES and the neuropsychological outcome. ESES is a kind of status epilepticus leading to nonconvulsive seizure symptoms hidden in sleep, manifested repeatedly during sleep, causing prolonged interference with cognitive functions [115], Recently it was shown by Tassinari et al. [116] that slow wave downscaling measuring according to amount of SWA, amplitude of slow waves, slope of waves, and amount of multipeak waves during night sleep is impaired in ESES patients. 

All three syndromes are characterized by a transient, age-dependent, nonlesional, and genetically based epileptogenic abnormality (however ESES may develop on the basis of structural damage as well). The brain tissue responsible for the disorder is localised around the Sylvian fissure and strongly correlated with speech function and cognition [117]. The interictal discharges are partially localized to this perisylvian area and partially show different degrees of generalization and secondary bilateral synchrony. Discharges in all syndromes appear in the form of a focal or generalized spike-wave pattern. The amount and persistence time of interictal discharges seem to correlate with the degree of cognitive deficits, and there is a correlation between the degree of spiking during sleep and the degree of cognitive deficits across syndromes. 

The epileptic discharges are accompanied with a slow wave component, associated with cognitive deficits rather than frequent seizures. This characteristic situation has been called “cognitive epilepsy.” The slow wave components of the GSW discharges may protect against conventional sustained, depolarization-based seizures but on the other hand interfere with normal cortical functioning. This assumption is strongly supported by several new results showing that sleep has a use-dependent homeostatic function, connected with sleep slow wave activity, needed for the plastic functions and impaired when abundant epileptic discharges interfere with it [118, 119]. 

*Lennox-Gastaut Syndrome (LGS) as a Developmental Epilepsy with Early Involvement of the Corticothalamic System.* LGS is an age-dependent syndrome beginning before the age of 4, but the termination is not clear. Patients may have a persistent syndrome during adulthood; there may be a “late onset LGS” as well [120–123]. The coexistence of several seizure types is typical. The most characteristic are tonic axial seizures occurring more frequently during NREM sleep, causing drop attacks in the wake state. Atypical absence with irregular GSW paroxysms is the other characteristic seizure form. The EEG shows frequent slow spike-wave paroxysms emerging from a slow and irregular background. Mental retardation is the rule. LGS is assumed to develop through “secondary generalization” based on early involvement of the thalamocortical network [124]. One of the most important features of LGS is that interictal and ictal epileptic manifestations appear during brain development suggesting that “excitation-producing etiologies and/or genetically induced aberrant cortical development and physiology during a sensitive window of enhanced epileptogenicity in the immature central nervous system induce synaptic remodelling leading to homotopic and thalamic epileptic discharge propagation and a diffuse bisynchronous epileptogenic process, with excess cortical excitation and increased corticothalamic oscillation” [124].

NREM sleep importantly activates slow GSWs, but their frequency does not reach the level seen in ESES. Another very characteristic feature of NREM sleep in LGS is the presence of runs of generalized paroxysmal fast activity (GPFA). The appearance of GPFA shows a strong correlation with deep slow wave sleep. These runs are bilaterally synchronous, with frontal predominance; their frequency is around 10 Hz. Very frequent in NREM, they never occur in REM and are rare in waking. Polygraphic recording reveals some ictal involvement in most of them. The most regularly observed somatic concomitant of the discharge is acceleration or deceleration of the heart- and respiration rate and increase of axial tone, most conspicuously in the neck muscles with a small elevation of the head and a little opening of the eye lids.

The slow GSW activity so characteristic for LGS has a frontal predominance and belongs to the category of secondary bilateral synchrony [125, 126]. This kind of pattern is held to be the result of focal epileptic activity propagating to the contralateral hemisphere through the corpus callosum, driving widespread corticothalamic entrainment [126].

The frequent occurrence of GPFA and their morphology raises the possibility of a relationship with sleep spindles. There is a relationship between the presence of slow spike wave discharges and GPFA. In our material the association of GSWD and GPFA was more than 90% in either ictal or interictal states.

In Doose syndrome where GPFA is not present, mental deterioration is less frequent and not as severe as in LGS [127, 128]. Recently we reported exceptional cases where typical paroxysmal fast activity was present without mental deterioration and intractability in difficult to treat IGE patients [129].

The influence of the frequent discharges mainly in sleep might have an effect on mental development. A possible effect could be interference with memory consolidation during NREM sleep [130]. Distorsion of physiological spindling by GPFA leading to giant pathological spindles may have a special worsening effect on memory consolidation. 

**Figure 6:**
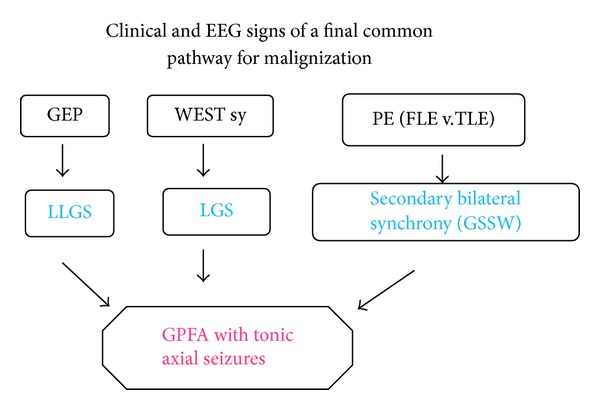
Schema of clinical and EEG signs of a final common pathway of malignization in three groups of patients: (1) West syndrome, (2) idiopathic generalized epilepsy, (3) certain frontal and temporal lobe partial epilepsies through secondary “Lennoxisation,” showing generalized fast paroxysmal activity usually in NREM sleep.

Earlier we worked out a concept interpreting GPFA as a final common pathway of malignization concept for primary and secondary generalized epilepsies [125] (Figure 6). Observing the paradoxical GPFA eliciting acute effect of benzodiazepine drugs and barbiturates we assumed an iatrogenic transformation of the GABA-chloride ionophor complex receptor structure by chronic use of these drugs in the treatment of these patients. The clinical impression that in the last 5–10 years the occurrence of GFPA is decreasing would support the hypothesis.

*Epilepsy with Unilateral or Bilateral Involvement of the Temporolimbic Network.* Temporolimbic network epilepsy (TLNE) is the most frequent epilepsy type in adulthood [131, 132]. The most frequent etiology is hippocampal sclerosis (HS) preceded by an “initial precipitatory insult” (atypical febrile seizure or status epilepticus) damaging the hippocampus and initiating an epileptogenic synaptic reorganisation in this structure, which may only lead to epilepsy after a long period (Mathern et al. 1996). Synaptic reorganisation of the hippocampal network is a key point in the evolution of temporolimbic epilepsies [133–135]. Other frequent etiological factors are tumours (gangliogliomas, embrioplastic neuroepitheliomas, and oligodendrogliomas), cortical dysgenetic malformations, cavernomas, and posttraumatic and postencephalitic lesions.

The main substrate of TLNE is held to be the hippocampus; however, more widespread temporal structural damage apparently plays a role [136, 137]. In the majority of temporolimbic epilepsies (TLNE) interictal EEG (mainly during sleep) or other diagnostic categories (neuropsychology, MRI, FDG-PET, MR spectroscopy, pathological work up) shows bilateral involvement and frequent contralateral spread of seizures.

 Complex partial and secondarily generalized seizures originating from the TLNE are frequently apparent during sleep as well as waking. Sleep seizures are more frequent in NREM sleep, and secondarily generalized seizures tend to be more prominent in sleep. Discharges in sleep appear in higher rate and in a more explicit form compared to the wake state findings. NREM sleep is associated with an increase of spiking rate, extension of electrical field, and the rate of bilateral independent discharges, while in REM sleep a restriction of the electrical field was observed [138–141]. Within NREM sleep the activation of temporal spiking was found to be the highest in stages 3-4 [139] increasing as patients move to deeper stages of NREM sleep [142]. Temporomesial compared to temporolateral cortical structures may display spiking at different sleep levels [143]. 

A small number of patients show activation during REM sleep, localizing the primary epileptogenic focus better than spike activity in either waking or NREM sleep. 

**Figure 7:**
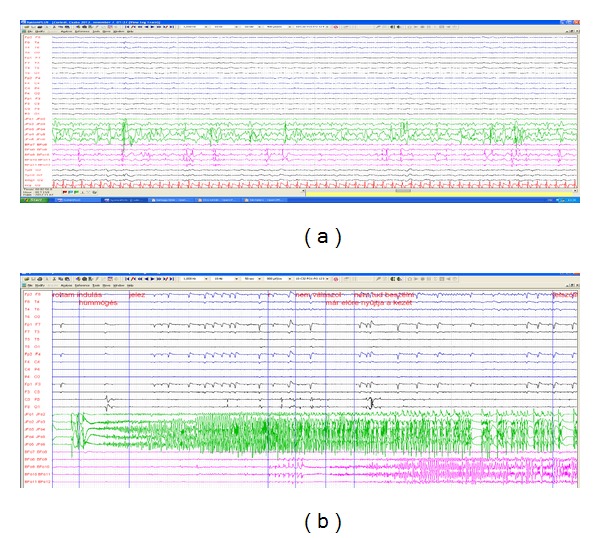
Interictal (a) and ictal (b) record of a patient with mediotemporal epilepsy investigated with foramen ovale (FO) electrodes picking up mediotemporal activity. (a) Intensive spiking in both FO electrodes independently with separate electromorphology, with abundance of slow waves in the right FO (green). Spiking is only sparsly reflected in the scalp electrodes. (b) Ictal recording shows seizure start that simultaneously with the clinical onset as sigma range oscillations in the right FO electrode contacts. Propagation to the contralateral side (purple) was seen several seconds later. Scalp electrodes show ictal activity only after propagation to the contralateral side in the FO, with theta range rhystmic activity. This record demonstrates the difficulties to verify the laterality relations in mediotemporal epilepsies without intracranial recordings, when both sides are involved.

Medial temporal lobe epilepsy has been conceptualized more and more as a spectrum of conditions showing a continuum between unilateral and bilateral involvement (Figure 7). Whether this continuum reflects a progressive course (secondary bilateralization) or determined purely by etiological constellations is still not enough clarified. The propensity to express bilateral discharges is particularly reflected in sleep records. The persistence of bilateral-independent interictal spiking in NREM sleep after surgery proved to be strongly associated with bad surgical outcome in our study [144].

The characteristic cognitive deficit conjoining TLNE is disturbance in declarative memory, due to hippocampal dysfunction which is in a certain extent side specific related to verbal memory in the dominant side and to visuospatial memory in subdominant hippocampal impairment. 

The role of hippocampus in memory consolidation in a dialogue with the cortex during NREM sleep, proposed firstly by Buzsáki [145], seems to be more and more studied in details and supported also in human beings [146–149]. It is plausible to assume that the memory impairment related to NTLE is underpinned by the epileptic interference with this process by hippocampal discharges, but direct proof of this is still lacking. There are only some lines of evidence for the role of epileptiform discharges during NREM sleep in the memory deficit interfering with hippocampal-cortical dialog [149].

*Nocturnal Frontal Lobe Epilepsy (NFLE) and the Prefrontal Mediobasal Network.* Since the early 1980s, a series of studies reported on patients with peculiar short motor seizures and dystonic-dyskinetic features, usually involving both sides of the body, clustering during NREM sleep, often accompanied with strange vocalisation, under different headings [150–152]. The attacks recur (with multiple episodes during the night) almost every night, at least in certain periods of the course of the disease. Consciousness is preserved or regained very soon. Approximately one-third of patients exhibit some kind of interictal epileptiform discharges (IED) localised to the frontal region. Ictal scalp EEG recording reveals epileptic features only exceptionally. The motor pattern is highly stereotyped for each individual with slight variation but may have highly variable patterns across patients. Almost half of the patients exhibit occasional GTCs in waking or sleep.

The seizures vary in the character of the hypermotor pattern. In some seizures the asymmetric tonic postural component is more prominent resembling those originating from the supplementary sensory-motor area (SSMA) region. The higher occurrence rate of SSMA seizure in sleep compared to wakefulness was demonstrated by Anan and Dudley [153].

In the mid 1990s Scheffer and coworkers [151] described a familial variant of NFLE with autosomal dominant inheritance (ADNFLE). 

Beside the association of NFLE seizures with sleep, there is also a characteristic link with NREM arousal dynamics. Seizures are always preceded by micro-arousals. Terzano et al. [154] demonstrated that motor events are closely related to periods of unstable NREM sleep and began during a CAP *A* phase. The overall CAP rate in his patients was increased, and when attacks were suppressed, the CAP rate decreased. In this kind of epilepsy the association of clinical (motor) epileptic events with NREM sleep arousal events is evident. 

In ADNFLE an epileptic sensitisation of the cholinergic arousal system has been found [18, 155]. This is underlain by ACh receptor mutations in different brain structures belonging to the arousal system [156]. The number of microarousals during NREM sleep increases in this type of epilepsy, and they release epileptic events [157]. Arousal disorder a kind of parasomnia in NREM sleep shows very similar symptoms both regarding the symptomatology of the nocturnal events and also regarding the interrelationship of the events with NREM micro-arousals. In addition there is a clear overlap in the occurrence of NFLE and arousal parasomnia within the patient's families [158].

 We propose that pathological arousals accompanied by confused behavior with autonomic signs and/or hypermotor automatisms are expressions of the frontal cholinergic arousal function of different degree, during the condition of depressed cognition by frontodorsal functional loss in NREM sleep. This may happen either if the frontal cortical Ach receptors are mutated in ADNFLE (and probably also in geneticaly not proved nonlesional cases as well), or without epileptic disorder, in arousal parasomnia, assuming gain in receptor functions in both conditions. 

**Figure 8:**
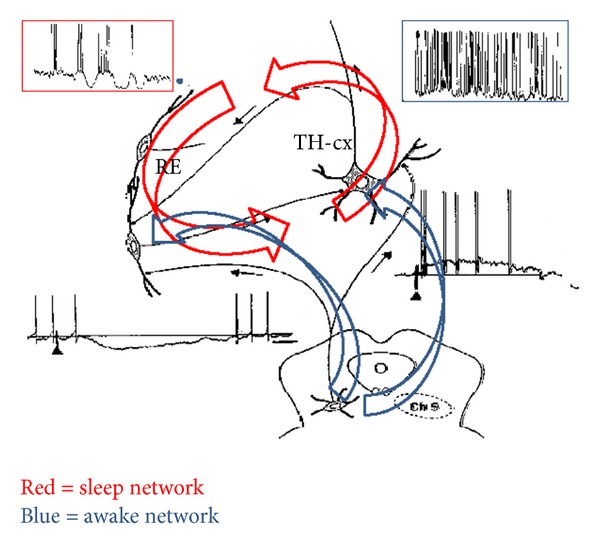
Schematic representation of arousal (blue) and sleep (red) network, according to Itier and Bertrand [18] using the Steriade's drowing [19]. In the inserts the thalamocortical transmitting mode is shown: in red frame the burst-firing mode during NREM sleep and in blue frame the tonic mode conveying continuous impulses from the thalamus to the cortex. The below other two inserts show how cholinergic arousal system inhibits the thalamic reticular activity which in the burst-firing mode provides recurrent inhibition of the thalamic relay neurons (big red arrow). The other influence of the arousal system fuels the cortical arousal through the thalamic relay cells.

Further we propose that NFLE and IGE represent epileptic disorders of the two antagonistic twin systems in the frontal lobe (Figure 8). NFLE is the epileptic facilitation of the ergotrop frontal arousal system, whereas absence of epilepsy is the epileptic facilitation of burst-firing working mode of the spindle and delta producing frontal thalamocortical throphotrop sleep system [159]. 

NFLE patients do not show much cognitive disturbances, neither ictally nor interictally. The lack of scalp EEG involvement is an interesting characteristic of NFLE, partially due to the localisation of epileptogenic areas in the hidden frontomedial and orbital surfaces. The interictal discharges do not show any GSW characteristics. This supports the assumption that the spike-wave generating thalamocortical system is not involved in NFLE. We have to assume that in NFLE arousal activation has direct access to frontal cortical circuitry involving the basal ganglia, responsible for hyperkinetic motor paroxysms. Greater involvement in motor functions contrasts with epilepsies related to the corticothalamic network, involved more in sensory information processing and cognition. Moreover a striking contrast can be established between the IGE and NFLE, while IGE may represent the epilepsy of the NREM sleep trophotrop network, the NFLE, and the ergotropic frontal cholinergic arousal/alarm network. 

## 2. Conclusions

**Figure 9:**
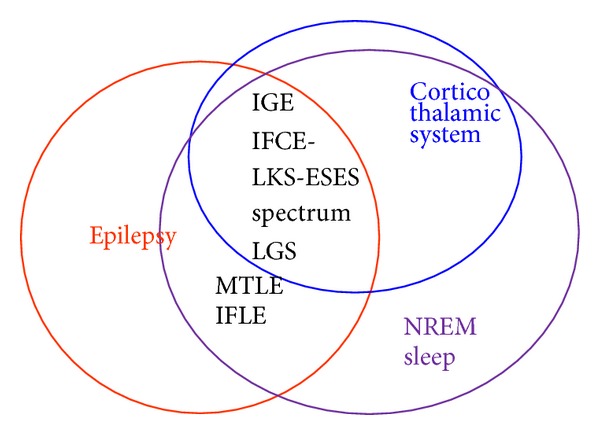
Relationship among the epilepsy networks, NREM sleep network, and the corticothalamic system.

Both clinical and EEG manifestations of many epilepsy syndromes linked to aspects of sleep and epileptic EEG manifestations in sleep are strongly associated with the presence of cognitive dysfunction (Figure 9). 

It is clear that in epilepsies involving the corticothalamic network like in IGE, the perisylvian network or LGS are harmful for cognition. Interference with cognition seems to be parallel to the amount and extension of SW discharges in NREM sleep. Discharges originating from one part of the thalamocortical associative areas can interfere with functions represented in that part of the network. For example, in LKS discharges in the perisylvian, mainly posterior part of the first temporal convolution interferes with certain aspects of speech function [160]. A more extensive functional deficit may be related to cortical disfacilitatory effects of the wave component of GSW discharges demonstrated by electrophysiological [161] and fMR methods [65]. A third component of cognitive impairment may be the interference of GSW discharges with the increasingly evidenced restorative functions of NREM sleep [118]. Therefore nowadays we have more and more evidence supporting the view that NREM sleep, and especially delta sleep homeostatic regulation, is governed by use-dependent plastic processes. In other words delta homeostasis and use-dependent plasticity are two different sides of the same coin probably representing the biological function of slow wave sleep [119, 162]. 

The occupation of a considerable amount of slow wave sleep by spike-wave discharges as in ESES and LKS or even in a more circumscribed way by rolandic discharges, and also in LGS, obviously interferes with restorative function, resulting in exhaustion of cognitive capacities. 

NFLE syndrome, without the entrainment of the SWD generator corticothalamic system, is relatively free from cognitive deficits that are in striking contrast with the well-known importance of the frontal lobe in cognitive functions.

In TLNE the development of secondary bilateral synchrony probably involves the thalamocortical system and may be responsible for certain cognitive decline, and the local involvement of hippocampal memory circuits interfere with the hippocampal dialog ensuring memory consolidation during sleep. In addition structural lesions due to the etiological factors (tumour, trauma, encephalitis, etc.) also may contribute to local cognitive dysfunctions. 

Activation during sleep is related to the network properties of the particular epileptic syndromes. So we can differentiate between several types of epileptic network activation by sleep: (1) via the thalamocortical system as main route and (2) other ways like (a) frontal epilepsy by epileptic transformation of the cholinergic arousal system in NREM sleep, (b) activation of temporolimbic epilepsy by NREM and REM sleep via hippocampal changes during sleep. 

Another aspect which has been mentioned during this work is that epileptic activation during NREM sleep is linked to phasic activation of reactive delta bouts. It is a global relationship with sleep valid for almost all epilepsies. In the majority of epilepsies interictal epileptiform discharges and also seizures are gated by CAP and within CAP A1 phase. In deep NREM sleep the up states of the bellow 1 Hz slow oscillation contain rich synaptic and HFO activity, while during down states disfacilitation is overwhelming. The alternation of these states with extremely opposite functionalities across alternations of up and down states might be also candidates to play important role in gating epileptiform activity.

## Abbreviations

IGE: Idiopathic generalized epilepsy
IFCE: Idiopathic focal childhood epilepsies
LKS: Landau-Kleffner syndrome
ESES: Electrical status epilepticus in sleep
LGS: Lennox Gastaut syndrome
MTLE: Medial temporal lobe epilepsy
IFLE: Idiopathic frontal lobe epilepsy.
